# Early Recognition of the PCL/Fibrous Carbon Nanocomposites Interaction with Osteoblast-like Cells by Raman Spectroscopy

**DOI:** 10.3390/nano11112890

**Published:** 2021-10-28

**Authors:** Aleksandra Wesełucha-Birczyńska, Anna Kołodziej, Małgorzata Świętek, Łukasz Skalniak, Elżbieta Długoń, Maria Pajda, Marta Błażewicz

**Affiliations:** 1 Faculty of Chemistry, Jagiellonian University, Gronostajowa 2, 30-387 Kraków, Poland; anka.kolodziej@doctoral.uj.edu.pl (A.K.); lukasz.skalniak@uj.edu.pl (Ł.S.); 2 Institute of Macromolecular Chemistry, Czech Academy of Sciences, Heyrovského Sq. 2, 162 06 Prague, Czech Republic; swietek@imc.cas.cz; 3 Faculty of Materials Science and Ceramics, AGH-University of Science and Technology, Mickiewicza 30, 30-059 Kraków, Poland; dlugon@agh.edu.pl (E.D.); mblazew@agh.edu.pl (M.B.); 4 Technolutions, Wiejska 7, 99-400 Łowicz, Poland; marynia85@gmail.com

**Keywords:** nanomaterials, poly(ε-caprolactone) (PCL), multi-walled carbon nanotubes (MWCNTs), electro-spun carbon nanofibers (ESCNFs), Raman microspectroscopy, human U-2 OS cell line, bioactivity

## Abstract

Poly(ε-caprolactone) (PCL) is a biocompatible resorbable material, but its use is limited due to the fact that it is characterized by the lack of cell adhesion to its surface. Various chemical and physical methods are described in the literature, as well as modifications with various nanoparticles aimed at giving it such surface properties that would positively affect cell adhesion. Nanomaterials, in the form of membranes, were obtained by the introduction of multi-walled carbon nanotubes (MWCNTs and functionalized nanotubes, MWCNTs-f) as well as electro-spun carbon nanofibers (ESCNFs, and functionalized nanofibers, ESCNFs-f) into a PCL matrix. Their properties were compared with that of reference, unmodified PCL membrane. Human osteoblast-like cell line, U-2 OS (expressing green fluorescent protein, GFP) was seeded on the evaluated nanomaterial membranes at relatively low confluency and cultured in the standard cell culture conditions. The attachment and the growth of the cell populations on the polymer and nanocomposite samples were monitored throughout the first week of culture with fluorescence microscopy. Simultaneously, Raman microspectroscopy was also used to track the dependence of U-2 OS cell development on the type of nanomaterial, and it has proven to be the best method for the early detection of nanomaterial/cell interactions. The differentiation of interactions depending on the type of nanoadditive is indicated by the ν(COC) vibration range, which indicates the interaction with PCL membranes with carbon nanotubes, while it is irrelevant for PCL with carbon nanofibers, for which no changes are observed. The vibration range ω(CH_2_) indicates the interaction for PCL with carbon nanofibers with seeded cells. The crystallinity of the area ν(C=O) increases for PCL/MWCNTs and for PCL/MWCNTs-f, while it decreases for PCL/ESCNFs and for PCL/ESCNFs-f with seeded cells. The crystallinity of the membranes, which is determined by Raman microspectroscopy, allows for the assessment of polymer structure changes and their degradability caused by the secretion of cell products into the ECM and the differentiation of interactions depending on the carbon nanostructure. The obtained nanocomposite membranes are promising bioactive materials.

## 1. Introduction

The most important task of regenerative medicine is to stimulate the body to carry out and accelerate the processes of self-repair of damaged cells and tissues [1,2]. The potential of regenerative medicine is related to the compilation of achievements in various fields, e.g., tissue engineering, genetics, biology, transplantology and materials engineering [1,3,4]. The task of material engineering is to design and manufacture substrates optimized to the needs and requirements of a given type of cells and tissue [5,6]. Therefore, materials planned for use in bone regenerative medicine should not only meet the conditions required for all biomaterials, i.e., biocompatibility, but also have the osteoinductive character and the ability to osseointegrate [7,8,9]. Another issue is to tailor the mechanical properties of the substrate to the natural bone parameters, and also biomimetically match at the macro, micro and nanoscopic level [10,11]. Natural substances such as collagen, cellulose, chitosan, alginic acid, bioceramics, biodegradable polymers and nanocomposites are readily used to prepare the bases of regenerative bone tissue [12,13,14,15,16,17].

PCL is a biocompatible resorbable material used in medicine, but its use is limited due to the fact that it is characterized by the lack of cell adhesion to its surface [10]. Various chemical and physical methods are described in the literature, as well as modifications with various nanoparticles aimed at giving it such surface properties that would positively affect cell adhesion [13]. The introduction of small amounts of nanoparticles in polymer matrices modifies the properties of polymers important for the applications in the field of regenerative medicine [18]. The type of nanoparticle introduced into the polymer matrix can cause not only a change in the polymer parameters, e.g., mechanical properties or thermal stability, but leads to a material with completely new properties, e.g., conductive or magnetic [19,20,21,22]. The adhesion of cells to the material surface depends on many factors, such as nano- and micro-scale topography, surface energy and certain mechanical properties—especially material stiffness, in particular—are the basic elements influencing the cellular response.

Carbon nanoforms (MWCNTs, CNFs, graphene) are materials with high potential for medical applications, not only in the area of their direct use (drug carriers, hyperthermia) but also in the field of surface or volume modifications of bioactive and biocompatible polymers [4,23,24,25,26]. Polymer nanocomposites containing carbon nanoparticles obtain a number of new functional properties that allow them to be used in nerve regeneration (nerve guide) or bone tissue (bioactive biomimetic scaffolds) applications [6,27,28]. The properties of polymer nanocomposites are closely related to the type of carbon nanoadditive and depend on the form, size and chemical structure of the surface [23,29,30]. Control of the properties of polymer nanocomposites due to their suitability for medical purposes requires a knowledge of phenomena at the molecular level accompanying the introduction of carbon nanoforms into the polymer matrix. We have carried out such tests for carbon layers on the titanium substrate [31,32], and we have also begun such studies for polymer nanocomposites [20]. It can be pointed out that the groups of atoms of the polymer matrix selectively interact with the nanoparticle of the nanoaddition.

In this work, nanomaterials in the form of membranes were produced by the introduction of multi-walled carbon nanotubes (MWCNTs) and functionalized multi-walled carbon nanotubes (MWCNT-f) as well as electro-spun carbon nanofibers (ESCNFs) and functionalized carbon nanofibers (ESCNFs-f) into a poly(ε-caprolactone) matrix (PCL). These nanomaterial membranes were brought into contact with the human osteoblast-like U-2 OS cell line and their interaction with the material was examined. The observed phenomena were compared with those observed for the reference polymer (PCL) membrane. The development of cell population, in the first days of culture, was monitored with fluorescence microscopy. Raman microspectroscopy was also applied to simultaneously verify interactions between the nanomaterials’ phases, i.e., at the interface of the fibrous carbon-based nanoparticles and polymer, and also at the nanomaterial/cell interface. The interaction was analyzed in relation to the changes observed in the crystallinity of the polymer matrix and carbon nanoparticles as well as the U-2 OS cell response. This study enriches the information obtained so far by applying two-dimensional correlation [33,34]. The Raman microspectroscopy method is regarded as one of the new analytical approaches to study liquid/solid interfaces at the molecular level [35].

In this study, we present research on a modified polymer with MWCNTs and we compare these results with a material modified with a completely different carbon nanoform, which is ESCNFs, i.e., a material different from MWCNTs both in terms of crystalline structure and geometric parameters. In our approach to the analysis of nanocomposite membranes, we use the possibility of insight into interactions at the molecular level between a complex nanomaterial, i.e., certain molecular fragments components of a polymer matrix or carbon nanostructure, with osteoblast-like cells using Raman microscopy. In other words, we unravel the chemical changes that take place in cells in contact with four types of materials and correlate them with changes occurring within nanocomposites, as well as characterizing the phenomena occurring in carbon nanoforms. It is clear that recognizing the molecular properties of materials is important because they influence their macroscopic characteristics.

## 2. Materials and Methods

### 2.1. Fabrication of Nanocomposite Membranes

**Figure 1:**
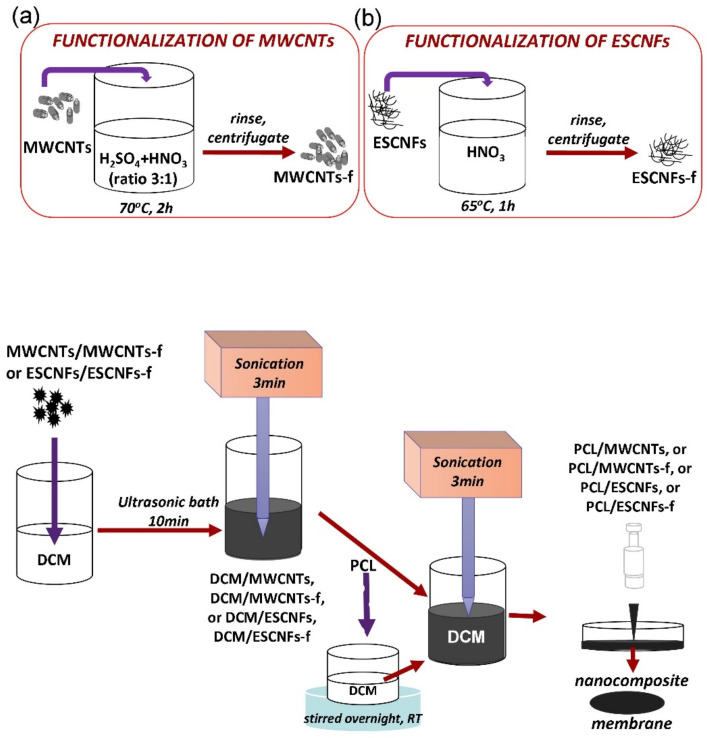
The nanocomposite membranes fabrication process; the inserts present a method of functionalization of carbon nanoadditives: (**a**) functionalization of MWCNTs; (**b**) functionalization of ESCNFs.

Poly(ε-caprolactone (PCL; (C_6_H_10_O_2_)_n_, Mn 45.000; purchased from Sigma-Aldrich, Warsaw, Poland), designed as a matrix, was dissolved in dichloromethane (DCM; provided by Avantor Performance Materials, Gliwice, Poland) to prepare its 10 wt% solution which was stirred overnight at room temperature. The nanoadditives, namely multi-wall carbon nanotubes (MWCNTs) or functionalized multi-wall carbon nanotubes (MWCNTs-f), electro-spun carbon nanofibers (ESCNFs) or functionalized carbon nanofibers (ESCNFs-f), were dispersed in an equal volume of organic solvent with an aid of the sonication process—firstly in an ultrasonic bath (L&R Manufacturing Co., Kearny, NJ, USA) for 10 min and then additionally by using a sonication probe for 3 min at an amplitude of 30% (BANDELIN electronic GmbH & Co. KG, Berlin, Germany).The obtained suspension was immediately transferred into the polymer solution. The mixture was sonicated for 3 min at an amplitude of 30% to ensure a good combination of both constituents, promptly poured onto a Petri dish (diameter 55 mm) and left at room temperature. The Petri dish was protected with punctured foil from too high rate of DCM evaporation. The produced polymer nanocomposites, PCL/MWCNTs and PCL/MWCNTs-f, PCL/ESCNFs or PCL/ESCNFs-f, contained 0.5 wt% nanoadditive in each material in relation to the weight of the polymer. The manufacturing process is shown in Figure 1, and was also described previously [33,34].

The MWCNTs (obtained from Nanostructured & Amorphous Materials, Inc., USA; purity: ≥95%; length: 0.5–2 μm; outside diameter: 10–30 nm) were functionalized in a mixture of sulphuric (VI) acid and 65% nitric (V) acid with 3/1 ratio 70 °C for 2 h (functionalized multi-walled carbon nanotubes, MWCNTs-f). Then, the nanotubes were rinsed with distilled water and centrifuged (Figure 1a) [20]. The total oxygen content in the MWCNTs is about 7%, which indicates a relatively small degree of functionalization of the tested nanotubes; for MWCNT-f it was estimated twice as much [36].

Carbon nanofibers were produced in the course of carbonization of a polyacrylonitrile (PAN) precursor, consisting of copolymers, 93–94 wt% acrylonitrile, 5–6 wt% methyl methacrylate and 1 wt% sodium allylsulfonate (purchased from Mavilon, Hungary). The PAN nanofibers were spun from a 11% solution N′N-dimethylformamide (DMF, acquired from Avantor Performance Materials Poland S.A.) using an electrospinning setup consisting of a high voltage generator (regulated from 1 to 20 kV), rotating tubular collector and a syringe with the polymer solution with a nozzle made of a stainless-steel needle with a diameter in the range of 0.6–1.2 mm. Prior to the process of electrospinning, the solution was stirred with a magnetic stirrer for 24 h. The average diameter of a nanofibers thus obtained was 250–280 nm. The as-obtained precursor was turned into carbon fibers in a three-step process. The first step was thermo-oxidative stabilization at a temperature of 250 °C and was performed for 1 h. The oxidation process was expected to transform the linear structure of the polymer into a cyclic structure. Then followed low-temperature (750 °C, 1 h) and high-temperature carbonization (1000 °C, 1 h) conducted in the protective atmosphere of nitrogen flow (30 L/h), with a heating rate of 5 °C per minute [37]. Carbon nanofibers were subjected to oxidation treatment in concentrated nitric acid (V) at 65 °C for 1 h (Figure 1b). Then samples were cooled down in the solution to room temperature, washed and dried in a dryer [38].

**Figure 2:**
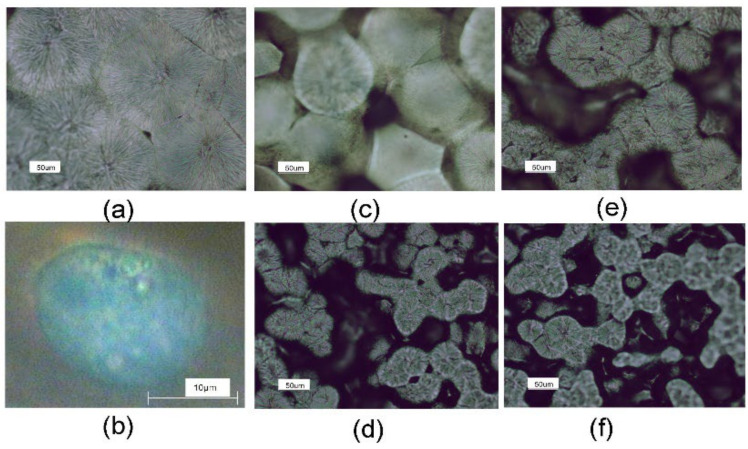
Microphotograph of the membrane top face: (**a**) PCL; (**c**) PCL/MWCNTs; (**d**) PCL/ESCNFs; (**e**) PCL/MWCNT-f and (**f**) PCL/ESCNFs-f, magnification 20×; (**b**) U-2 OS human cell on PCL/MWCNTs on the first day of culture, immersion objective, magnification 60×.

The obtained membranes are shown in Figure 2a,c–f.

### 2.2. Contact Angle Measurements and Surface Free Energy Evaluation

The contact angle measurements were performed on a SAM10Mk1 (KRÜSS GmbH, Germany) goniometer using deionized water, by the sessile drop method. In order to determine the surface free energy (SFE) contact angle for non-polar diiodomethane (CH_2_I_2_) was measured, additionally to water (the polar liquid). A calculation model according to Owens, Wendt, Rabel and Kaelble (OWRK-model) available for SFE within the factory-supplied software was employed. The calculation requires the contact angles of two liquids with known polar and diffuse SFE fractions. Then, the free energy of a surface can be considered as composed of the polar part and the dispersed part. At least ten contact angle measurements in different locations on the surface were performed to obtain an average value. The results of the contact angle measurements and surface free energy for all membranes were statistically analyzed by calculating the arithmetic mean of the results and the standard population deviation function in Excel software. The Kolmogorov–Smirnov test of normality was performed, *p* < 0.001.

### 2.3. Cell Culture

The human U-2 OS cell line (ECACC, cat. no. 92022711, lot no. 10K035) is one of the first generated cell lines from the moderately differentiated osteosarcoma and is used quite frequently to test materials bioactivity [39]. The cells were cultured in Mc Coy’s medium (BioWest, Nuaillé, France) supplemented with 10% Fetal Bovine Serum (FBS, BioWest, Nuaillé, France). The cells were grown at 37 °C in a humified atmosphere containing 5% CO_2_.

The monoclonal population of cells with stable expression of maxFP-Green, a tailored green fluorescence protein, was developed from the U-2 OS cell line by transfection with the pmaxFP-Green-N vector (Amaxa Biosystems, Cologne, Germany). The transfection was completed using Lipofectamine 2000 (Life Technologies, Carlsbad, CA, USA), and stable clones were selected with G418 (Life Technologies). The resulting clones were picked, repopulated and verified for the maxFP-Green expression with the flow cytometry. A clone, designated U-2 OS-Green, had optimal expression of a transgene and was used for the experiments.

The nanomaterial samples were sterilized in 70% ethanol for 30 min. After washing three times with saline phosphate buffer solution (PBS), they were exposed to UV light for 30 min. For the experiments, the cells were seeded at a relatively low density (10,000 cells per cm^2^) in 12-well plates. The next day, the materials were transferred into new 12-well plates with a fresh cell culture medium, in order to exclude the cells growing on the plastic from imaging. The procedure was described previously [33,34].

### 2.4. Fluorescence Microscopy

The development of the fluorescent U-2 OS-Green on the analyzed nanomaterials was monitored using a Leica DM IL Led fluorescence microscope (Leica Microsystems, Wetzlar, Germany), equipped with a Leica DFC3000 G digital camera(Leica Microsystems, Wetzlar, Germany). The images were captured with Leica Application Siute X 3.3.3.16958 software using the Leica N PLAN 10x/0.25 PH1 objective, and analyzed with ImageJ 1.48v [40]. While the cells were seeded and grown on the upper surface of the materials, just before the imaging the materials were inverted, and inverted back after the imaging for further culture. The growth of the cells was monitored at the 1st, 2nd, 3rd and 6th day post-seeding and every day the images were captured using the same camera settings (40 ms exposure, gain = 1) to enable quantification of the fluorescence intensity (day 6 was an exception, when the 15 ms exposure was performed to capture the properly exposed image, but this was compensated in the calculations).

**Figure 3:**
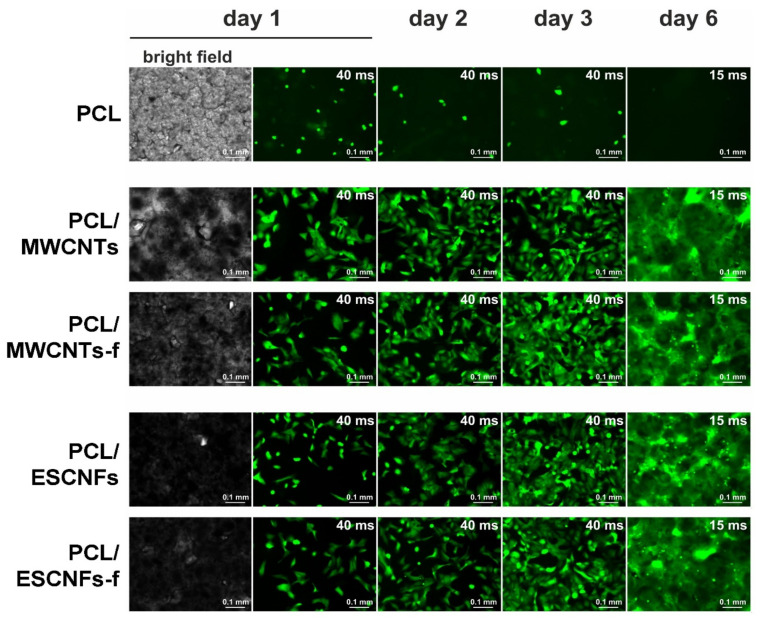
U-2 OS-Green cells expressing green fluorescent protein (GFP) in bright field and cultured for the 1st, 2nd, 3rd (40 ms exposure, gain = 1) and 6th day post-seeding (15 ms exposure, gain = 1), from top: PCL; PCL/MWCNTs, PCL/MWCNTs-f, PCL/ESCNFs and PCL/ESCNFs-f; mag. 10× (original images were published in [33,34]). The exposure time on the sixth day was reduced due to the appropriate capture of the exposed image. The cell growth experiments were performed at least three times for each material.

**Figure 4:**
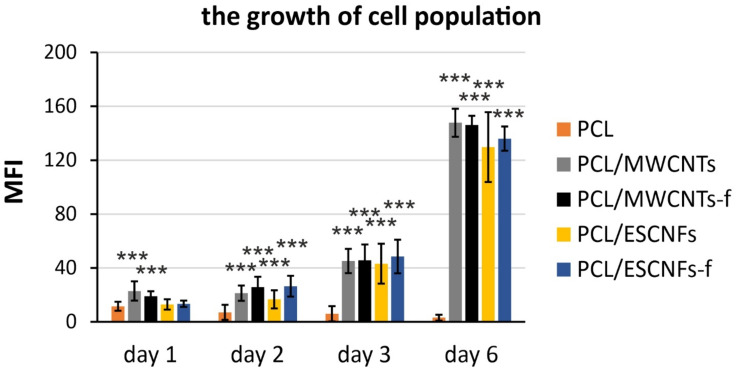
The growth of U-2 OS-Green cells on PCL; PCL/MWCNTs, PCL/MWCNTs-f, PCL/ESCNFs and PCL/ESCNFs-f; nanomaterials, monitored at the 1st, 2nd, 3rd (40 ms exposure, gain = 1) and 6th day post-seeding (15 ms exposure, gain = 1). The bar represents the means ± SD of the mean fluorescence intensities (MFI) from 9–20 separate images (median images number = 12.5). For the MFI quantifications background subtraction was applied. Statistical analysis was performed with one-way ANOVA with the Tukey post-hoc test: ***, *p* < 0.001 versus PCL. The differences in exposure time were compensated in the calculations.

The information on the number of photomicrographs was analyzed and the statistical analysis is provided in the captions in Figure 3 and Figure 4. For fluorescence quantification, background subtraction was performed for each individual image in order to provide more adequate data and better reflect the differences observed in the photomicrograph.

### 2.5. Raman Microspectroscopy

A Renishaw inVia spectrometer (Wotton-under-Edge, Gloucestershire, UK), working in a confocal mode, connected to a Leica microscope (Leica Microsystems, Wetzlar, Germany), was used for the measurement of the Raman spectra. The beam from a 785 nm HP NIR (high power near IR) diode laser was focused on the samples by a Nikon immersive objective 60× magnifying (NA = 0.5). Raman light was dispersed by diffraction grating with 1200 grooves/mm. Laser power was kept low, c.a. 1–3 mW on the sample, to ensure minimum disturbances of the samples. The Raman spectra of the studied PCL/MWCNTs/ MWCNTs-f and PCL/ESCNFs/ ESCNFs-f nanomaterial membranes and reference PCL membrane cultured with U-2 OS cells were collected at the 1st, 3rd, 6th and 8th day post-seeding. Measurements of membranes with cells seeded on their surface were recorded in the range of 2000–400 cm^−1^ to shorten the measurement time to reduce cell signal disturbance. Four accumulations were made for each measurement site. The spectra were averaged by adding five spectra to thereby also improve the signal-to-noise ratio. Statistical analysis of Raman spectra was carried out in the OMNIC program, the average position and the standard deviation resulting from the summation.

Factory-supplied software was used to preprocess, i.e., cosmic spike removal, smooth and a baseline corrected (Renishaw, WiRE v. 2.0 and 3.2). The height, a width and percentage of the Lorentz–Gauss curve were fitted. From matching, the band parameters, the positions of the component bands, its height, full width at half-height (FWHH) and their area were determined. The curve fit procedure allowed for the analysis of changes in the marker areas characterizing regions of the matrix polymer chains, carbon nanoadditives and cells, through appropriate band intensity ratios. Changes could be determined by comparison with reference spectra and the corresponding reference intensity ratios for: the polymer matrix, nanocarbon additive and cell. Statistical analysis was performed with PCA with Calibration 99.30505%; Validation 97.97677% (the first measurement day); Calibration 97.08938%; Validation 95.18859% (the third measurement day); Calibration 99.35546%; Validation 98.39545% (the sixth measurement day); Calibration 97.8542%; Validation 94.61504% (the eighth measurement day).

## 3. Results and Discussion

### 3.1. The Morphology of Membranes of PCL with Fibrous Carbon Nanoparticles

The micrographs taken from the top face of the nanocomposite membranes and reference PCL membrane are shown in Figure 2. The main factor limiting the growth of a single spherulite is the growth of other spherulites in its immediate vicinity. The well-formed spherulites typical for PCL polymer of radius ~95 μm (Figure 2a) become smaller along with functionalization. Their radius is equal to ca. 70, 40, 25 and 23 μm for PCL/MWCNTs, PCL/MWCNTs-f, PCL/ESCNFs and PCL/ESCNFs-f, respectively. Additionally, significant changes in the surface morphology of the polymeric membranes are observed (Figure 2b–e). On the basis of the microphotographs it can be assumed that both unmodified fibrous carbon nanoforms and also, respectively, functionalized nanoparticles constitute the nucleation centers when introduced into the polymer matrix solution. The simultaneous crystallization of spherulites in many sites, combined with a limited possibility of their recrystallization, leads to the formation of numerous pores. As a consequence, the spherulitic structure of the material gradually disappears along with the increasing number of heterogeneous seeds of the crystallization.

### 3.2. Contact Angle Measurements and Surface Free Energy (SFE)

Wettability was determined at room temperature. Although the introduction of fibrous carbon nanoparticles into the polymer matrix resulted in a slight decrease in the nanomaterial membranes hydrophobicity, the calculated values of the contact angle for the tested materials are quite similar. Based on previous research. the values of the wetting angle for the top membranes surface are equal to 94.7 ± 1.2; 88.8 ± 1.3; 90.6 ± 3.7, 89.5 ± 1.5, 89.4 ± 1.2 for PCL, PCL/MWCNTs, PCL/MWCNTs-f, PCL/ESCNFs and PCL/ESCNFs-f, respectively [41]. The interaction of the liquid phase with the materials occurs through polar and dispersion forces. SFE values are relatively large compared to other polymeric materials, but comparable for the tested membranes. Interestingly, the polar SFEs components for PCL/MWCNTs/MWCNTs-f are slightly higher than that for PCL/ESCNFs/ESCNFs-f, Table 1. Surface energy is the result of many factors; however, it cannot be ruled out that in this case the surface morphology will have a key impact on the parameters of the analyzed materials [4]. Perhaps the size of the spherulites affects the properties of the materials.

### 3.3. The Comparison of Growth of U-2 OS Cells on the Membranes of PCL with Fibrous Carbon Nanoparticles

To test the ability of the materials to serve as a substrate of cell growth, human U-2 OS-Green cells were seeded on the tested nanomaterials, PCL/MWCNTs, PCL/MWCNTs-f and PCL/ESCNFs, PCL/ESCNFs-f, and also as a reference on the PCL membrane [33,34]. The cells were seeded at a relatively low confluency and cultured in standard cell culture conditions. This procedure is used to assess the properties of materials in contact with living cells [24,25,26,27,28,29,30,31,32,33,34,35,36]. It is common practice to use human osteogenic sarcoma cells (e.g., U-2 OS) cultured in vitro to investigate the biocompatibility of materials [37]. The growth of the cells was monitored by fluorescence microscopy at the 1st, 2nd, 3rd and 6th day post-seeding.

As we have described in our previous studies, for all of the studied nanocomposite substrates, a marked increase in the cell population was observed in the first week of culture while for a reference PCL membrane no such increase was observed [33,34]. In this manuscript, the cell growth was compared between all four nanocomposite membranes and the PCL in a single analysis (Figure 3). The growth was estimated by employing a quantitative method in fluorescence microscopy, i.e., quantifying mean fluorescence intensities for every captured image, which is proportional to the cell population numbers, calculated as the mean pixel intensity [42]. The analysis revealed a clear, exponential increase in the number of cells, starting from the second day of the culture, and observed on the consecutive days (Figure 4). This increase was estimated, by comparing the first and sixth day of culture, as equal to 3.5, 4.8, 3.9 and 4.0 for PCL/MWCNTs, PCL/MWCNTs-f, PCL/ESCNFs and PCL/ESCNFs-f, respectively. This suggests outstanding proliferation of the cells on the tested nanomaterials. In contrast, the numbers of cells seeded on a reference PCL membrane even slightly decreased with time, which may partially be a consequence of cell detachment during the material inverting procedure performed for microscopic visualization, and suggests that the modification of the PCL surface with nanoforms of carbon significantly improves the cells’ attachment to the materials (Figure 4).

### 3.4. Raman Microspectroscopic Analysis of the Membranes of PCL with Fibrous Carbon Nanoparticles/Cells Interactions

**Figure 5:**
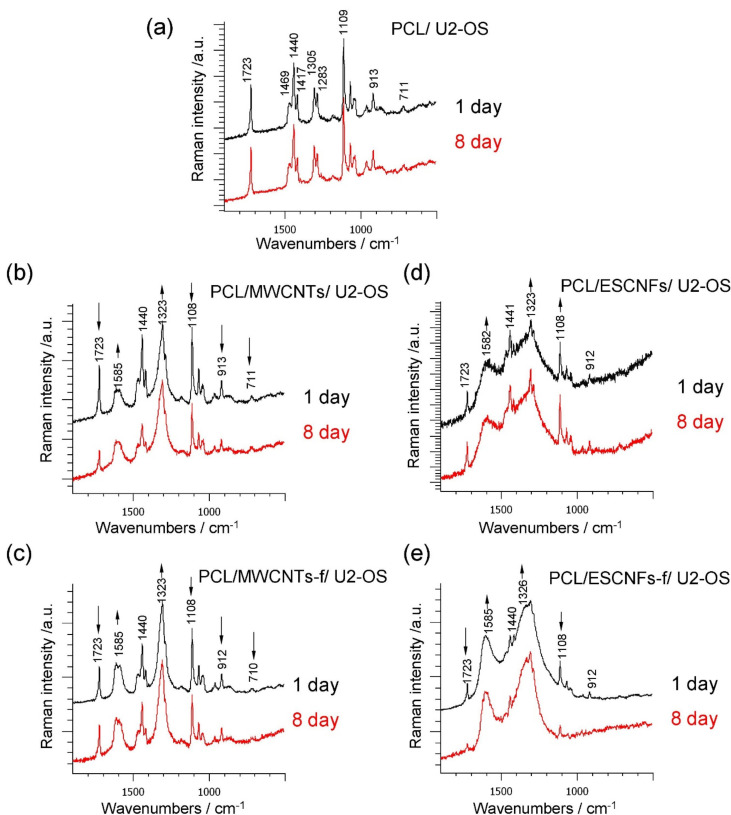
Raman spectra of the U-2 OS cell culture on the substrate: (**a**) PCL; (**b**) PCL/MWCNTs; (**c**) PCL/MWCNTs-f; (**d**) PCL/ESCNFs and (**e**) PCL/ESCNFs-f, on day 1st and 8th; 1900–500 cm^−^^1^ range, 785 nm excitation line.

Raman spectroscopy was used to monitor the interactions of polymer (PCL)-based carbon, fibrous nanomaterial membranes with human osteoblast-like U-2 OS cells at the 1st, 3rd, 6th and 8th day post-seeding. This interaction was monitored by analyzing changes in the crystallinity of the polymer matrix, by an identification of the ordering of the respective carbon nanoforms and recognition of the osteoblast U2-OS cells’ marker bands. Figure 5 shows the Raman spectra on the first and last day of the experiment.

#### 3.4.1. PCL Matrix Crystallinity

**Figure 6:**
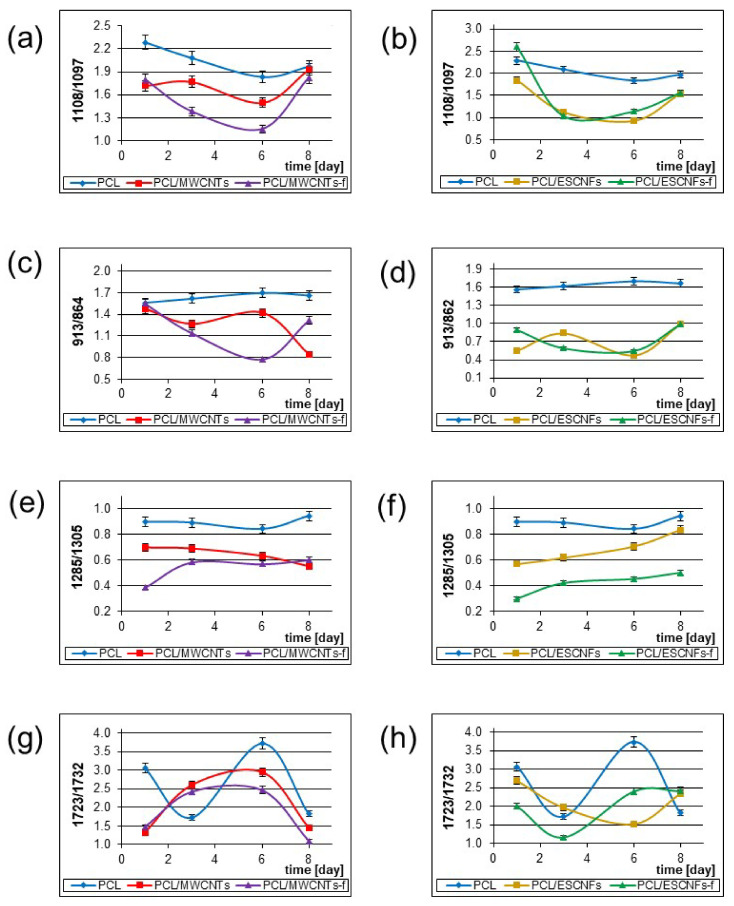
The intensity ratio (integral) of the PCL marker bands characterizing the crystallinity of the tested polymer nanomaterials constituting the substrate for U-2 OS cell culture, in the following days of culture. For PCL, PCL/MWCNTs, PCL/MWCNTs-f: (**a**) 1108/1097 cm^−1^ (Statistical analysis with PCA with Calibration 99.8774%; Validation 99.50629%); (**c**) 913/864 (Statistical analysis with PCA with Calibration 99.12821%; Validation 93.47666%); (**e**) 1285/1305 (Statistical analysis with PCA with Calibration 99.94765%; Validation 99.68526%); (**g**) 1723/1732 (Statistical analysis with PCA with Calibration 99.97593%; Validation 99.81823%) and for PCL, PCL/ESCNFs, and PCL/ESCNFs-f: (**b**) 1108/1097 (Statistical analysis with PCA with Calibration 99.8774%; Validation 99.6284%); (**d**) 913 /864 (Statistical analysis with PCA with Calibration 99.7179%; Validation 97.28348%); (**f**) 1285/1305 (Statistical analysis with PCA with Calibration 99.94656%; Validation 99.65956%); (**h**) 1723/1732 (Statistical analysis with PCA with Calibration 99.98973%; Validation 99.85501%), variance is defined by y-axis error bars, OMNIC software.

The observed significant Raman bands and their assignments are collected in Table 2. The intensity of some marker bands characterizing the polymer crystallinity, i.e., stretching vibrations at 1723 cm^−1^ due to the ν(C=O), 1108 cm^−1^ band assigned to ν(COC), 913 cm^−1^ to ν(C-COO), and also the deformation vibrations at δ(CH_2_) at 1440 and 1417 cm^−1^, marked with arrows in Figure 5b–e, changed significantly in the first days of the culture. The evolution of the changes taking place in the tested nanomaterials in the consecutive measurement days was assessed in relation to selected markers of the polymer crystallinity, by matching lines and analytically determining the component bands in the appropriate ranges, and presented in Figure 6 [20,43].

The intensity ratio of 1108 (cryst)/1097 (amorph) cm^−1^ ν(COC) vibrations in the PCL chain decreases in the first days of culture for all types of membranes, which indicates a decrease in the crystallinity of the polymer matrix, and then its increase on day 8 (Figure 6a,b). The increase in crystallinity on the 8th day of culture seems to indicate the stabilization of the polymer matrix in the process of cell adhesion related to their intense proliferation. It cannot be ruled out that the increase in proliferation may affect the growth of the band contribution of about 1100 cm^−1^, for lipids and DNA, O-P-O backbone stretching, although this band for U-2 OS is not very intense in our measurement conditions [34,51,55]. The characteristics of the adjacent spectral regions give the intensity ratio of 913 (cryst)/864 (amorph) cm^−1^ due to ν(C-COO) vibrations, which for PCL/MWCNTs-f is similar to the previous ones, but for the PCL/MWCNTs it decreases in consecutive days (Figure 6c). However, for the PCL/ESCNF and PCL/ESCNF-f crystallinity of the polymer matrix it does not change significantly (Figure 6d). Variability in the C-C region appears to indicate that cell adhesion is taking place.

The band at 1305 cm^−1^ due to ω(CH_2_) comes from the crystalline and amorphous PCL domains, while 1285 cm^−1^ originates only from ω(CH_2_) in crystalline areas. The intensity ratio of 1285/1305 cm^−1^ decreases for PCL/MWCNTs, while it increases for PCL/ESCNF and PCL/ESCNF-f (Figure 6e,f). Interaction with cells seems to influence this process. The oscillation range ω(CH_2_) indicates an increase in the interaction of PCL/ESCNFs and PCL/ESCNFs-f with cultured cells, observed by a systematic increase in the amorphousness in the studied system [15]. The influence of proliferating cells cannot be excluded, so that the Pro signal of approx. 1280 cm^−1^ increases the intensity ratio 1285/1305 [45].

Another important parameter of crystallinity of the polymer matrix is the intensity ratio of 1723 (cryst)/1732 (amorph) cm^−1^ reflecting involvement of the C=O group in interactions with cells, which grows for PCL/MWCNTs and PCL/MWCNT-f, but at the 8th day significantly decreases (Figure 6g). It is different for PCL/ESCNF, for which the intensity ratio decreases and then increases, while for PCL/ESCNF-f the behavior is directly opposite (Figure 6h).

A decrease in the 1723/1732 intensity ratio, observed for PCL/MWCNTs membrane, indicates a decrease in the crystallinity of the polymer matrix in the area of C=O groups of the polymer chain, on the 8th day of culture. This indicates the influence of cells on their growth substrate, which occurs through the enlargement of the amorphous domains in the material. This evaluation is consistent with the results of a two-dimensional correlation analysis [34].

A similar pattern, the reduction of I1723/I1732 intensity ratio, was observed for the PCL/MWCNTs-f membrane. This feature indicates the increasing amorphous nature of the polymer matrix in contact with U-2 OS cells. These results are consistent with the relationship determined by 2D-COS [34].

For the next type of nanomaterials, for PCL/ESCNFs and PCL/ESCNFs-f membranes, on the 8th day of culture, an increase in crystallinity was observed for both types of membranes. Two-dimensional correlation spectroscopy indicates the participation of carbon nanostructures in interactions with cells [33]. The increase in the I1723/I1732 intensity ratio seems to be related to the structure of nanofibers that interact with cells in a different way [38].

**Figure 7:**
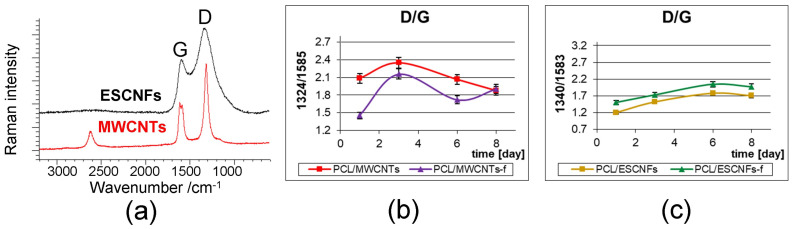
(**a**) Raman spectra of MWCNTs and CNFs nanoadditives (reference spectra), in the range 3200–500 cm^−^^1^; the I(D)/I(G) intensity ratio for: (**b**) PCL/MWCNTs, PCL/MWCNTs-f; (Statistical analysis with PCA with Calibration 99.86237%; Validation 99.14962%) (**c**) PCL/ESCNFs and PCL/ESCNFs-f, (Statistical analysis with PCA with Calibration 99.86559%; Validation 99.01482%) polymer nanomaterials constituting the substrate for U-2 OS cell culture, in the following days of culture; 785 nm excitation line, variance is defined by y-axis error bars, OMNIC software.

The relative increase in the intensities of the above-mentioned bands indicates an increase in amorphicity in the studied nanomaterials, in comparison to the reference PCL membrane for which changes almost do not happen (Figure 7). The observed trend can be correlated with the increase in the population of the cells, whose strong development in the subsequent days of the culture was monitored in fluorescence microscopy (Figure 3), which modifies the extracellular matrix and induces changes observed on the upper surface of the membranes.

#### 3.4.2. The Arrangement of Carbon Nanostructures

In Figure 7a Raman spectra of carbon nanostructures, MWCNTs and ESCNFs, are shown. The Raman spectra contain, in the first order region, the G- and D-band at ca. 1590 and 1330 cm^−1^, respectively. A characteristic parameter determining the ordering in carbon materials is the ratio of the intensity of D-band and G-band [54,56,57]. Plots reflecting the changes of this parameter on consecutive measurement days were collected for PCL/MWCNTs and PCL/MWCNTs-f and for PCL/ESCNFs and PCL/ESCNFs-f, respectively, in Figure 7b,c. For both types of carbon nanotubes in the PCL matrix, MWCNTs and MWCNTs-f, the I_D_/I_G_ crystallinity parameter fluctuates in the first days of cell culture, reaching some stabilization and similarity after eight days. Carbon nanofibers, ESCNFs and ESCNFs-f, are characterized by a systematic increase in disorder in polymer membranes and, interestingly, achieve a similar value after eight days of cell culture, such as carbon nanotubes. This indicates a slightly different process of cell adhesion depending on the carbon nanoadditive used. It also indicates the changes taking place in the nanomaterial itself and the modifications that nanoadditives undergo during cell culture; see Figure 3 and Figure 4.

#### 3.4.3. Raman Spectroscopy of U-2 OS Cell Development on PCL Membranes with Fibrous Carbon Nanoparticles

**Figure 8:**
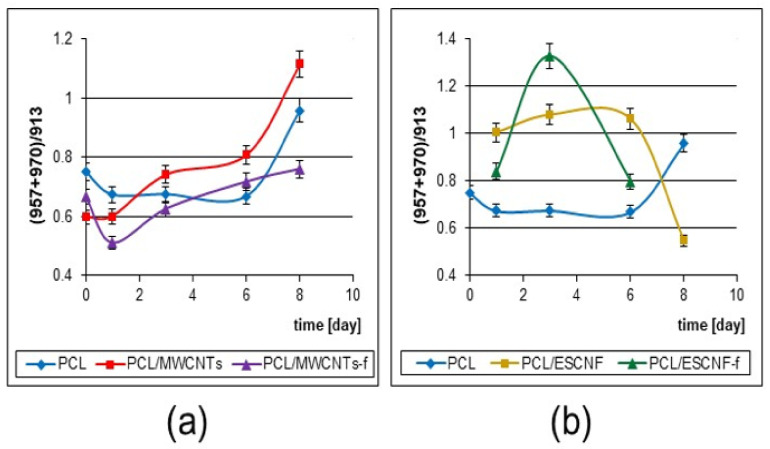
The intensity ratio (integral) of marker bands (957 + 970)/913 cm^−1^ characterizing the growth and development of collagen for U-2 OS cells cultured on: (**a**) PCL, PCL/MWCNTs and PCL/MWCNTs-f (Statistical analysis with PCA with Calibration 98.61861%; Validation 95.60048%); (**b**) PCL, PCL/ESCNFs and PCL/ESCNFs-f (Statistical analysis with PCA with Calibration 99.90554%; Validation 99.07312%), variance is defined by y-axis error bars, OMNIC software.

U-2 OS cells have specific growth characteristics. Less than 50% of the cells are positive for collagen type I, however, positive labeling was found for molecules related to the cartilage such as collagen types II, IV, V and X [58]. The labeling profile for the U-2 OS cells remains constant and does not depend on cell density, so these osteoblastic markers, after secretion into the extracellular matrix (ECM), may be visible in the Raman spectra. In different types of collagen structures one can anticipate the presence of Gly, because this amino acid is every third residue. Actually it is monitored in the Raman spectra as visible bands of 711 and 913 cm^−1^ (Figure 5; Table 2) [44,59]. It seems convincing to pay attention to the integral intensity in the 975–930 cm^−1^ range that increases, and indicates an increase in Proline content in the ECM (Figure 8) [45] Building collagen: proline and hydroxyproline are its essential amino acid components, and can represent in some domains up to 28 and 38%, respectively [59]. 

The cell growth on the studied materials is very good (Figure 3), however, the cells adhesion monitored by Raman spectroscopy proceeds in a different way, possibly due to the presence of the nanoparticle (Figure 6). The formation of the extracellular matrix may justify its influence and the observed pattern in the first days of culture, even if the U-2 OS cell line, like all osteosarcoma cell lines, shows a very heterogeneous labeling profile, which also affects the kinetics of its proliferation [58]. 

Types II, IV, V and X collagen show positive labeling for the U-2 OS cells line [58]. Collagen II is fibrous, the protein comprises a righthanded bundle of three parallel, left-handed polyproline II-type helices [46,59]. Type IV collagen belongs to the basement membranes and the form supramolecular networks that control cell adhesion, migration and differentiation [47]. Type V collagen is a minor component of the collagen fibrils with type I collagen [48]. The presence of type V collagen in the vicinity of the basement membranes and in the collagen fibers suggests that it can act as a linker and can also contribute to the fibril structure. Type V collagen characteristics indicate that this molecule regulates the development, differentiation and tissue repair of extracellular matrix organization [49]. The short chain of collagen X provides a pericellular matrix during ossification [50]. After secretion into the ECM, these molecules further interact to form higher supramolecular organizations that interfere with 3-dimentional nanocomposites’ support. These categories of collagen include the fibrillar and network-forming proteins. They blend in very well with the structure of the nanocomposite membranes and may provide structural support for the cells and tissues.

From the second point of view the cellular metabolism products lead to progressive degradation of the membrane arrangement that is especially visible for the nanocomposite PCL/ESCNFs membrane (Figure 8b). A slightly larger number of functional groups on the carbon nanotube seems to lead to the formation of polymer matrix–MWCNTs-f nanocomposite as a tightly intertwined mat, the degradation of which is not as fast as PCL/ESCNFs. However, both these types of carbon nanotubes, MWCNTs and MWCNTs-f, and both types of carbon nanofibers, ESCNFs and ESCNFs-f, seem to very efficiently stimulate the growth of cells (Figure 4 and Figure 8).

### 3.5. Morphology of U-2 OS Cells Growing on PCL Membranes with Fibrous Carbon Nanoparticles

**Figure 9:**
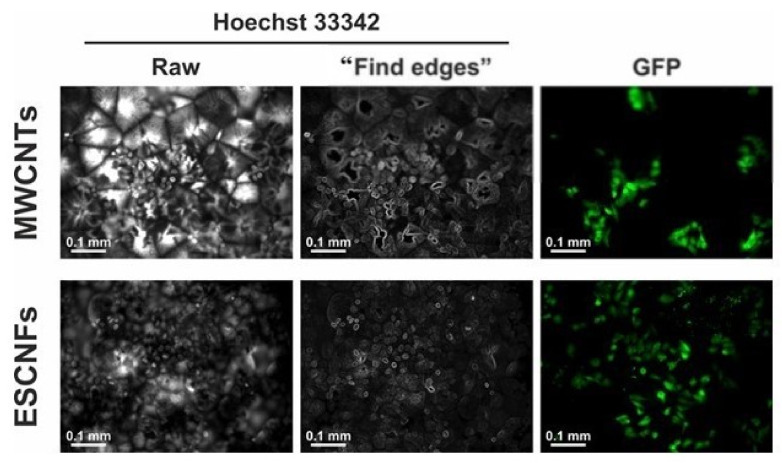
U-2 OS-Green cells distribution on PCL/MWCNTs and on PCL/ESCNFs on the 2nd day of culture. The specimens were live-stained with Hoechst 33342 to visualize cell nuclei. As shown in the “Raw” column, Hoechst stained not only cell nuclei but also the material (white signal, left panels). Thus, a “find edges” algorithm was used to visualize the shape of cell nuclei (the central panels). Right panels present GFP signal from the cells.

Fluorescent images taken on the second day after seeding revealed the cells of elongated shape, which is characteristic for adherent cells growing on a cell culture surface (Figure 3) [58]. In order to further verify the initial condition of the seeded cells, at the second day of the culture the cells were stained with Hoechst 33342 fluorescent dye, which selectively binds to dsDNA molecules and stains cells nuclei. At that point, it became clear that the Hoechst 33342 stains and also the PCL revealed the hidden nanotopography of the material (Figure 9). Following the staining, the PCL micelles were clearly visible as bright areas, separated by dark grooves. A deeper look into the Hoechst-stained cell nuclei revealed no signs of necrotic disruption of the nuclei or apoptotic nuclear fragmentation/blebbing. Instead, the cells contained round-shaped, undisrupted nuclei, suggesting good condition of the cells growing on both PCL/MWCNTs materials.

Just following the seeding (day 2), the cells tended to be located between the PCL micelles, as revealed by the Hoechst 33342 staining (Figure 9). This is presumably due to the nanotopography of the material surface, which easily allows the cells to settle in the grooves between the micelles before they adhere to their surface [10]. However, in time, while the population of the cells grew, the cells also colonized the surfaces of the micelles. This was evident after 6 days of the culture, when almost 100% of the material surface was covered by the cells (Figure 3). Interestingly, the cells were distributed almost evenly on the membrane with the second type of nanoadditive, ESCNFs. Moreover, the cells adapted to the underlying nanocomposite membranes to form the three-dimensional, thick specimens, penetrating deeper into the membrane pores. This was visible as intense, blur and scatter images of the cells (Figure 3).

## 4. Conclusions

Based on the fluorescence microscopy and Raman microspectroscopic data, the effect of carbon nanoadditives on the polymer structure and usefulness to stimulate the growth of bone tissue and cartilage were determined. Fluorescence microscopy that monitors the nanocomposite materials containing carbon nanoforms, PCL/MWCNTs and PCL/MWCNTs-f, as well as PCL/ESCNTs and PCL/ESCNTs-f culture with human U-2 OS cell line have shown that depending on the type of functionalization and geometric parameters of the nanoaddition, they have the bioactivity properties required for materials intended for bone tissue regeneration. The Raman spectroscopy analysis demonstrated that the degradation mechanism occurred mainly in the amorphous domains of PCL and resulted in increased polymer crystallinity, which is compatible with other reports [60]. The degradation of the membrane arrangement depends on the nanoadditive. Selected spectroscopic markers allow us to approximate the complex phenomena occurring at the interface polymer/carbon nanoaddition/U-2 OS model cell. In the current work we present research on a modified polymer with MWCNTs and we compare these results with a material modified with a completely different carbon nanoform, which is ESCNFs, i.e., a material both in terms of crystalline structure and geometric parameters different from MWCNTs. Secondly, we use an approach in which we use Raman in a way that is different from commonly applicable procedures. At the same time, we want to show which chemical changes take place in cells in contact with four types of materials and correlate them with changes occurring within nanocomposites, as well as characterize the phenomena occurring in carbon nanoforms.

Modern spectroscopic methods are therefore a significant support for other analytical methods, already in the first days of cell culture on nanomaterial. The obtained nanocomposites are promising bioactive materials for bone and cartilage tissue engineering. It should be noted that the presented materials require further assessment in accordance with applicable regulations in the context of their potential use in applications in contact with a living organism.

## Figures and Tables

**Table 1:** Contact angle for diiodomethane and surface free energy for the top surfaces (the contact surface with the U-2 OS cells) of the studied membranes.

Material	Contact Angle for Diiodomethane [°]		Surface Free Energy [mN/m]		Disperse Part [mN/m]		Polar Part [mN/m]	
	Value	StDev	Value	StDev	Value	StDev	Value	StDev
PCL	29.31	2.76	45.83	1.42	44.51	1.12	1.32	0.29
PCL/MWCNTs	26.58	4.80	47.66	2.36	45.57	1.80	2.09	0.56
PCL/MWCNTs-f	25.98	3.07	46.70	1.60	45.83	1.13	0.88	0.48
PCL/ESCNFs	29.31	3.20	45.93	1.62	44.51	1.30	1.43	0.32
PCL/ESCNFs-f	35.71	3.02	42.88	2.02	41.70	1.42	1.19	0.60

**Table 2:** Observed characteristic Raman bands [cm^−1^] and their assignments for PCL, PCL/MWCNTs, PCL/MWCNTs-f and PCL/ESCNFs and PCL/ESCNFs-f nanocomposite membranes in the first day of culture with human U-2 OS cell line, 785 nm laser line.

Raman Bands [cm^−1^]					Assignment
PCL	PCL/ MWCNTs	PCL/ MWCNTs-f	PCL/ ESCNFs	PCL/ ESCNFs-f	
712 ± 1	712 ± 1	713 ± 2	713 ± 1	713 ± 2	δ(CH_2_), δ(NH_2_), Gly; CS, Cys [44,45,46,47,48,49,50,51]
865 ± 1	862 ± 1	862 ± 2	861 ± 1	861 ± 2	ν(C-COO) PCL (amorph); o.o.p. δ(CH2), Pro; collagen [20,43,45,52]
913 ± 1	913 ± 1	912 ± 1	912 ± 1	912 ± 1	ν(C-COO), PCL (cryst); τ(CH_2_)&τ(NH_2_), Gly; collagen [20,43,44,52]
958 ± 1	958 ± 1	957 ± 1	958 ± 1	958 ± 1	ν(C-COO), PCL; ring str., Pro [20,43,45]
1038 ± 1	1038 ± 1	1037 ± 1	1038 ± 1	1037 ± 1	ν(COC), PCL; ω(CH_2_), Pro; ν(CN)&ν(CC), Gly [20,43,44,45]
1064 ± 1	1064 ± 1	1064 ± 1	1064 ± 1	1064 ± 1	ν(COC), PCL (amorph) [20,43]
1109 ± 1	1109 ± 1	1108 ± 1	1109 ± 1	1108 ± 1	ν(COC), PCL (cryst); collagen [20,43,52]
1284 ± 1	1284 ± 1	1284 ± 1	1283 ± 1	1283 ± 1	ω(CH2), PCL (cyst); δ(CH2), Pro [20,43,45]
1305 ± 1	1305 ± 1	1305 ± 1	1305 ± 1	1304 ± 1	ω(CH2), PCL (cryst and amorph) [20,43]
-	1323 ± 1	1323 ± 1	1340 ± 2	1341 ± 2	D1-disorder-induced A_1g_ mode in graphite plane; δ(CH_2_), Pro [45,53,54]
1418 ± 1	1418 ± 1	1418 ± 1	1418 ± 1	1418 ± 2	δ(CH2), PCL; γ(CH2)), Gly [20,43,44,52]
1441 ± 1	1441 ± 1	1441 ± 1	1441 ± 1	1441 ± 1	δ(CH2), PCL (cryst.); δ(CH2), Pro [20,43,45]
1469 ± 1	1468 ± 1	1467 ± 1	1466 ± 1	1470 ± 1	δ(CH2)), PCL; collagen [20,43,52]
-	1587 ± 1	1585 ± 1	1584 ± 1	1585 ± 1	corresponding to G-graphite tangential mode [30,31,53]
-	1615 ± 1	1614 ± 1	1615 ± 1	1614 ± 1	D2-band due to due to in-plane defects and heteroatoms [54]
1723 ± 1	1723 ± 1	1723 ± 1	1724 ± 1	1723 ± 1	ν(C=O), PCL (cryst) [20,43]
1732 ± 1	1733 ± 1	1733 ± 1	1732 ± 1	1723 ± 1	ν(C=O), PCL (amorph) [20,43]

## Data Availability

All data generated or analyzed during this study are included in this published article. Moreover, the datasets used and/or analyzed during the current study are available from the corresponding author on reasonable request.
